# On Hazardous Pills for Weight Loss and Cysticercosis

**DOI:** 10.4269/ajtmh.22-0196

**Published:** 2022-05-31

**Authors:** María Teresa Galán-Puchades, Màrius V. Fuentes

**Affiliations:** Parasites & Health Research Group, Department of Pharmacy, Pharmaceutical Technology and Parasitology, Faculty of Pharmacy, University of Valencia, Burjassot-Valencia, Spain

Dear Sir,

We have read with curiosity, as well as with alarm, the article by Zhang et al. on a human case of disseminated cysticercosis because of the intentional ingestion of *Taenia* eggs by a woman with the aim of losing weight.^1^ Tapeworms have been marketed as a weight loss product for over 100 years, and despite the known health risks, tapeworms continue to be advertised and sold today. Dieters swallow beef tapeworm cysticerci (*Cysticercus bovis*, the *Taenia saginata* metacestode), usually in the form of a pill. The theory is that the tapeworms will reach maturity in the intestine, and that the adult stage will absorb food. This could cause weight loss, along with possible symptoms such as diarrhea and vomiting.

According to the case report, the patient did not ingest any cysticerci, but rather the eggs of *T. saginata* contained in the pills. If the eggs were viable, they would turn into cysticerci, but only in intermediate hosts, cattle in the case of *T. saginata*. Therefore, these eggs would not have any effect in humans, which was the reason why the authors did not find any reference to the ingestion of *Taenia* eggs to lose weight. Likewise, the authors speculated that the ingested eggs could be *T. solium*, rather than *T. saginata,* as the patient developed disseminated cysticercosis, and among human *Taenia* tapeworms only the eggs of *T. solium* are infective for humans.^1^ In these speculations, *T. asiatica*, a species whose ability to cause human cysticercosis is still unknown,^2^ was not considered. If the egg providers identified the source of these eggs (*T. saginata* adult stage) through the morphology of the gravid proglottids, it should be considered that *T. asiatica* has the same gravid proglottid morphology as *T. saginata. Taenia asiatica* is present not only in China (where the case is reported) but also in many other Asian countries,^3^ although the country of origin of the eggs is apparently unknown. Likewise, *T. asiatica* cross-reacts with *T. solium* in the ELISA the authors used.^4^ Therefore, a molecular analysis of the patient’s cysticerci would have been necessary to assess the etiology of this case of disseminated cysticercosis. As no specific diagnostic technique was conducted, the possibility that *T. asiatica* was responsible should not be ruled out.

It would be hard to believe that the providers of the pills purposely supplied *T. solium* eggs for human consumption, considering the risk of dying of neurocysticercosis, as happened in an illustrative case with death after the intentional ingestion of *T. solium* eggs published in 1994.^5^ Thus, considering the consequences apparently derived from the ingestion of the pills the patient purchased, a molecular analysis of these eggs would have been desirable to take legal action against the company in case the eggs were *T. solium,* to prevent possible fatal cases of neurocysticercosis in other pill consumers.
